# Tabes Dorsalis With Spinal Meningeal Involvement and Possible Concomitant Epstein–Barr Virus Reactivation

**DOI:** 10.7759/cureus.112327

**Published:** 2026-07-09

**Authors:** Huong T Nguyen

**Affiliations:** 1 Neuroscience, Vinmec Times City International General Hospital, Hanoi, VNM; 2 Health Sciences, VinUniversity, Hanoi, VNM

**Keywords:** ebv-positive, epstein-barr virus, iv ceftriaxone, spinal pachymeningitis, syphillis, tabes dorsalis

## Abstract

We report an exceptionally rare case of tabes dorsalis with concomitant hypertrophic spinal pachymeningitis and incidental qualitative detection of Epstein-Barr virus (EBV) DNA in the cerebrospinal fluid (CSF), highlighting the diagnostic challenges and potential systemic complications of late neurosyphilis. A 44-year-old immunocompetent man presented with a two-year history of progressive sensory ataxia. Thoracic spine magnetic resonance imaging demonstrated longitudinal posterior column T2 hyperintensity with diffuse dural thickening and enhancement. Initial multiplex polymerase chain reaction of the CSF detected EBV DNA, raising concern for viral myelitis and prompting consideration of antiviral therapy. However, serological testing confirmed syphilis (rapid plasma reagin (RPR) 1:16; Treponema pallidum hemagglutination assay (TPHA) 1:20,480), while HIV and Human T-lymphotropic virus-1 (HTLV-1) testing were negative. Although CSF-Venereal Disease Research Laboratory (VDRL) was unavailable, the diagnosis was revised to probable neurosyphilitic myelopathy with tabetic features based on the clinical presentation, serological findings, CSF profile, and characteristic neuroimaging abnormalities. Intravenous ceftriaxone was initiated, resulting in clinical improvement, reduced CSF pleocytosis, and clearance of EBV DNA on follow-up analysis, suggesting incidental viral reactivation rather than active EBV infection. The patient was discharged in stable neurological condition but died suddenly within 24 hours. Although the exact cause of death could not be established, a cardiovascular complication of late-stage syphilis was considered the most likely explanation. This case broadens the recognized imaging spectrum of neurosyphilis by demonstrating hypertrophic spinal pachymeningitis with incidental EBV DNA detection and highlights the importance of considering syphilis in patients with posterior column myelopathy. It also emphasizes the need for comprehensive cardiovascular assessment in patients with late-stage syphilis to identify potentially life-threatening systemic complications.

## Introduction

Syphilis is a sexually transmitted infection (STI) caused by the spirochete Treponema pallidum. Tabes dorsalis, a late neurosyphilis manifestation, causes sensory ataxia, neuropathic pain, and sphincter dysfunction from demyelination of the posterior columns of the spinal cord and dorsal roots [1]. Abnormal magnetic resonance imaging (MRI) findings are uncommon; when present, they typically manifest as longitudinal T2-weighted hyperintensities in the dorsal spinal columns, whereas marked dural thickening and enhancement mimicking hypertrophic spinal pachymeningitis is rarely observed [2]. Furthermore, chronic inflammation, particularly STIs, can reactivate latent viruses such as Epstein-Barr virus (EBV) [3]. In the antibiotic era, tabes dorsalis is rare, mainly reported in immunocompromised individuals. We report a rare case in an immunocompetent patient diagnosed with probable tabes dorsalis with radiologically-defined dural involvement and Epstein-Barr virus DNA detection.

## Case presentation

A 44-year-old male patient was referred to the hospital from a local clinic due to a two-year history of progressively worsening lower limb numbness and weakness. Symptoms started approximately two months after experiencing a flu-like illness. Initially, he experienced intermittent numbness in the left lower extremity, with similar symptoms involving the right lower extremity emerging six months later. Over subsequent months, he developed progressive lower limb weakness, gait unsteadiness, and frequent falls, notably exacerbated with eye closure, eventually requiring the use of a wheelchair. He also reported numbness in the anterior chest wall. There was no history of bowel or bladder dysfunction, visual impairment, skin rash, weight loss, or sicca symptoms. He had been diagnosed with type 2 diabetes mellitus one month prior, which was well-controlled, and had concurrently ceased smoking. He denied alcohol consumption. The initial referral documentation from the local clinic noted no high-risk behaviors, as a detailed sexual history had not been obtained at that stage.

**Figure 1:**
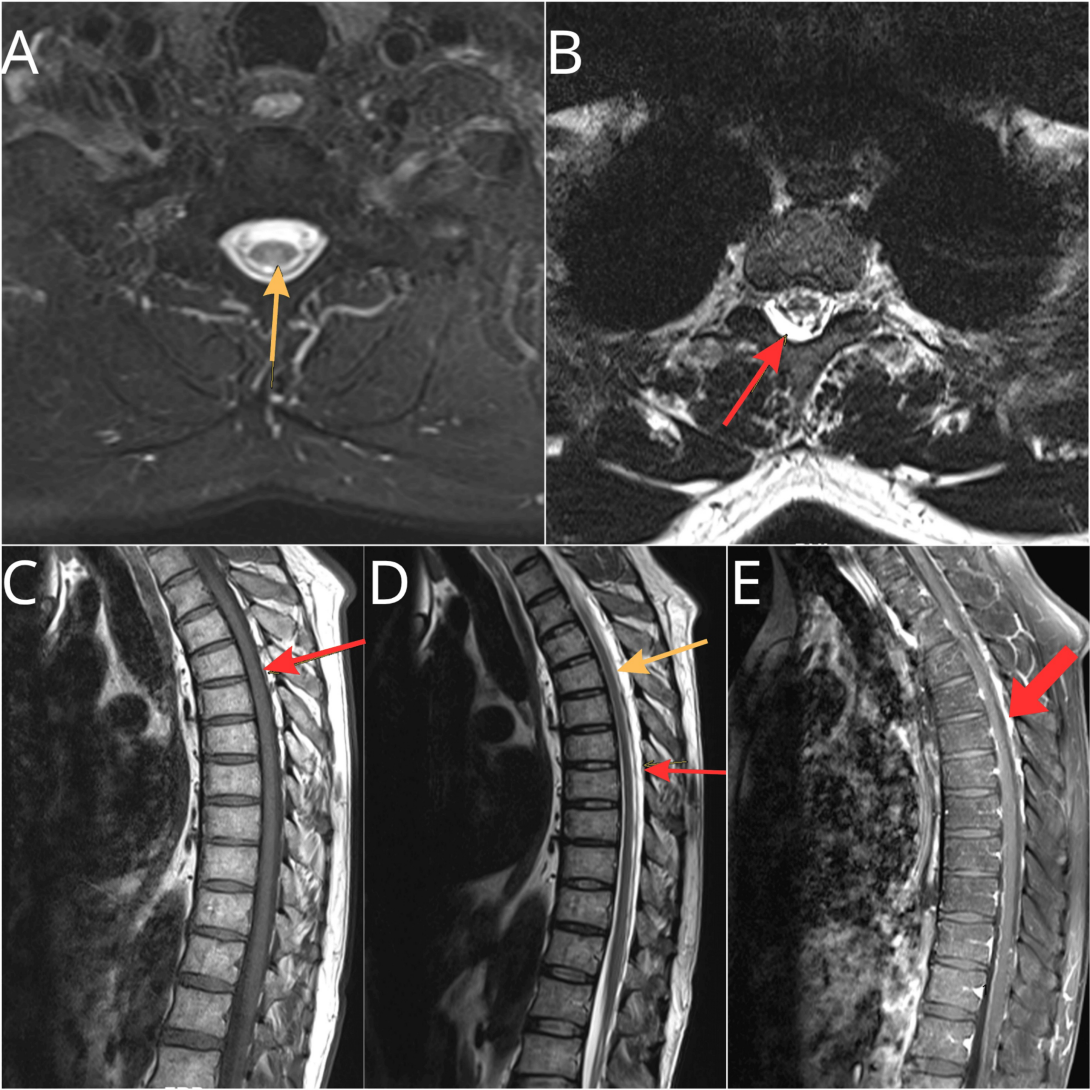
Magnetic resonance imaging of the thoracic spine Axial T2-weighted (T2W) image at T4 (A) and sagittal T2W image at T3–T8 (D) reveal hyperintensity of dorsal columns (yellow arrow). Axial T2W image at T6 (B) demonstrates a subdural fluid collection (red arrow). Sagittal T1-weighted (T1W) image (C) and T2W (D) reveal thickening of the dorsal dura mater (red arrow). Sagittal contrast-enhanced T1W image (E) shows marked enhancement of the thickened dorsal dura mater (bold red arrow).

Thoracic spine MRI demonstrated diffuse T2 hyperintensity in the posterior spinal cord from T3 to T8 without contrast enhancement, along with dural thickening and enhancement, and a heterogeneous epidural fluid collection at the same levels (Figure 1).

Diffusion-weighted imaging (DWI) was not performed.

Given the absence of prior trauma, spinal surgery, or signs of systemic infection, this collection was radiologically interpreted as an inflammatory exudate reactive to the underlying syphilitic pachymeningitis rather than an epidural abscess or hematoma. The patient's laboratory findings are presented in Table 1.

**Table 1:** Laboratory parameters at baseline and during early antibiotic therapy WBC, white blood cell; TPHA, Treponema pallidum hemagglutination assay; RPR, rapid plasma reagin; CSF, cerebrospinal fluid; HIV, human immunodeficiency virus; HTLV-1, human T-cell lymphotropic virus type 1; EBV, Epstein–Barr virus; VZV, varicella-zoster virus; CMV, cytomegalovirus; AQP4, aquaporin-4 antibody; MOG, myelin oligodendrocyte glycoprotein antibody; IIFT, indirect immunofluorescence test; PCR, polymerase chain reaction.

Investigation	Before antibiotics	Day three of antibiotic therapy	Reference range
Blood tests			
WBC (×10⁹/L)	11.1	8.3	4-10
Neutrophils (%)	70.4	66.7	42.8-75.8
Hemoglobin (g/L)	141	138	130-160
Platelet count (×10⁹/L)	354	301	150 - 450
Creatinine (µmol/L)	51.8	-	59-104
Glucose (mmol/L)	12.1	8.6	3.9-5.6
Homocysteine (µmol/L)	5.58	-	0-15
Vitamin B12 (pmol/L)	158.7	-	25.1-165.0
Serum AQP4-IgG (IIFT)	Negative	-	Negative
Serum MOG-IgG (IIFT)	Negative	-	Negative
TPHA titer	Positive (1/20480)	-	Negative
RPR titer	Positive (1/16)	-	Negative
HIV	Negative	-	Negative
HTLV-1	Negative	-	Negative
EBV IgM	Negative	-	Negative ( <0.5)
EBV IgG	Positive (66.12)	-	Negative ( <0.75)
Cerebrospinal fluid analysis			
WBC (/µL)	180	53	<5
Lymphocytes (%)	75	80	
Protein (g/L)	0.75	0.57	0.15-0.45
Glucose (mmol/L)	7.61	5.7	2.2-3.9
CSF/serum glucose ratio	0.62	0.66	>0.6
Chloride (mmol/L)	127	-	120-130
CSF culture	Negative	-	Negative
EBV PCR	Positive	Negative	Negative
HSV PCR	Negative	Negative	Negative
VZV PCR	Negative	Negative	Negative
CMV PCR	Negative	Negative	Negative
Mycobacterium tuberculosis PCR	Negative	-	Negative
Non-tuberculosis mycobacteria PCR	Negative	-	Negative

Cerebrospinal fluid (CSF) analysis showed pleocytosis (180 cells/µL, 75% lymphocytes) and elevated protein (0.7 g/L). EBV DNA was detected by multiplex polymerase chain reaction (PCR) in the CSF, while Herpes Simplex Virus (HSV), Varicella-Zoster Virus (VZV), and Cytomegalovirus (CMV) were negative. Serum EBV IgG was positive, and IgM was negative. Based on these findings, a presumptive diagnosis of EBV-associated meningitis was made, and the patient was transferred to our hospital for intended intravenous administration of antiviral and corticosteroids.

Upon examination, he exhibited superficial sensory loss in a dermatomal pattern from T5 to T8 and both lower extremities from the thighs distally, more pronounced in the distal segments. Vibration and joint-position senses were absent in the distal lower extremities, and the Romberg sign was positive. Lower limb strength was 3/5 proximally and 4/5 distally. Deep tendon reflexes were absent in both legs but preserved in the upper limbs. Babinski signs were negative, and sphincter control was intact. Nerve conduction studies (NCS) demonstrated preserved conduction in motor and sensory nerves of all four limbs, slightly prolonged F-wave latencies in lower limb nerves, and absent Hoffmann reflexes. Further detailed history taking elicited a report of unprotected sexual intercourse with a sex worker approximately 10 years prior. Serological testing for syphilis was performed, revealing a positive Treponema pallidum Rapid Plasma Reagin (RPR; titer 1:16) and a positive Treponema pallidum Treponema Pallidum Haemagglutination Assay (TPHA; titer 1:20480). HIV antibodies and Human T-lymphotropic virus type 1 (HTLV-1) were not detected. 

To evaluate for potential underlying immunosuppression in the context of EBV detection, further workup was performed. The patient had no history of prior corticosteroid or immunosuppressive therapy. A peripheral blood smear, total lymphocyte count, chest X-ray, and abdominal ultrasound revealed no evidence of hematological disorders or occult malignancy. Subsequent serological testing for autoimmune etiologies, including aquaporin-4 and myelin-oligodendrocyte glycoprotein antibodies, was negative. Anti-nuclear antibody was negative, and homocysteine and vitamin B12 levels were within normal limits.

Given the positive syphilis serology, the clinical presentation of myelitis, and MRI findings, the diagnosis was revised to tabes dorsalis. Due to the unavailability of aqueous crystalline penicillin G at our hospital, treatment was initiated with intravenous ceftriaxone 2 g every 24 hours as an alternative regimen, with a planned total duration of 14 days. Clinically, the patient reported subjective improvement in presternal numbness, but no significant improvement in gait disturbance was noted.

Repeat CSF analysis three days after antibiotic initiation showed a decrease in cell count (53 cells/µL) and protein (0.57 g/L); EBV DNA was no longer detected by polymerase chain reaction (PCR) at this timepoint. The patient received a total of four doses of intravenous ceftriaxone over four consecutive days at our hospital. Following the fourth dose, he was discharged with a plan to complete the remaining course at the local hospital. However, that institution subsequently reported that the patient had died out of hospital from unknown causes later that same day, prior to the administration of the next scheduled antibiotic dose. No autopsy was performed at the request of the deceased's family.

## Discussion

Our case presented with a classic clinical picture of tabes dorsalis, characterized by a gradual onset of sensory ataxia correlated with posterior thoracic column lesions on MRI, alongside positive syphilis serology. Notably, the patient had no prior history of syphilis diagnosis or treatment. In this context, the high RPR titer (1:16) strongly supported active neurosyphilis rather than a serological scar from a past episode. Beyond the classic posterior column involvement, the MRI also demonstrated hypertrophic spinal pachymeningitis at the thoracic level. While leptomeningeal enhancement of the spinal cord is well-documented, occurring in up to 55% of thoracic neurosyphilis cases [4], dural lesions are rarely reported and are typically associated with spinal gummas [5]. Other common etiologies of hypertrophic spinal pachymeningitis, such as tuberculous pachymeningitis or meningeal carcinomatosis [6], were considered highly unlikely given the negative CSF testing for tuberculosis, the absence of malignant cells, and the lack of history regarding trauma or prior lumbar puncture. Although we cannot definitively prove that the spinal dural lesion was directly caused by Treponema pallidum, a plausible explanation is that the intense, chronic inflammation within the posterior cord secondarily extended to involve the adjacent meninges.

Another important finding in this case was the presence of EBV DNA in the CSF, which initially led colleagues at the referring hospital to suspect EBV myelitis. The patient's clinical features and MRI appearance, however, were inconsistent with the central cord-predominant lesions typical of EBV myelitis, prompting us to consider alternative diagnoses, including neurosyphilis. Although CSF Venereal Disease Research Laboratory (VDRL) testing was not available in our laboratory, the combination of RPR antibody titers of 1:16 and TPHA of 1:20480, along with posterior column signs, led us to establish a diagnosis of probable neurosyphilis/tabes dorsalis and initiate antibiotic rather than antiviral therapy. As the patient requested early discharge to complete the remaining course of intravenous antibiotics at a local hospital near his home, a discharge plan was established. Consequently, a repeat lumbar puncture was performed one day prior to discharge (on day three) to obtain a preliminary assessment of treatment response and acute inflammatory trends. This early follow-up confirmed the appropriateness of our approach, showing a reduction in leukocyte count and the clearance of EBV DNA. Given the patient's immunocompetent state, the transient presence of EBV likely represented localized viral reactivation driven by the chronic inflammation of the active Treponema pallidum infection, which may compromise local T-cell surveillance. Crucially, the clearance of EBV DNA achieved solely through antibiotic therapy, without antiviral intervention, further underscores that this reactivation was an incidental, inflammation-dependent phenomenon rather than the primary driving pathology. We chose ceftriaxone over penicillin G, which was unavailable, based on several reports showing equivalent efficacy in improving clinical outcomes and CSF parameters [7]. While tabes dorsalis is a late stage of syphilis and treatment may not lead to significant neurological recovery, establishing the etiologic diagnosis allowed us to avoid unnecessary or prolonged therapies.

The unanswered question in this case remains the cause of the patient's sudden death. No Jarisch-Herxheimer reaction was observed during the antibiotic infusion. He returned home in a stable cognitive and psychological condition, without signs of acute infection, and with no history of medication or stimulant abuse. According to his relatives, the patient experienced a sudden loss of consciousness and cardiac arrest while resting in bed. Given the staging of his disease, cardiovascular complications of late-stage syphilis, such as a thoracic aortic aneurysm rupture or myocardial infarction, could be considered in the differential diagnosis [8]. However, because cardiovascular syphilis requires comprehensive clinical confirmation and no objective cardiovascular evaluation or autopsy was performed, the definitive etiology remains speculative. Nonetheless, this tragic outcome underscores the clinical importance of considering large-vessel imaging and thorough cardiovascular screening during the evaluation of patients with late-stage neurosyphilis.

## Conclusions

This report describes a rare presentation of tabes dorsalis associated with hypertrophic spinal pachymeningitis and incidental EBV DNA detection in the CSF. The case illustrates how neurosyphilis can mimic inflammatory or infectious myelopathies, creating significant diagnostic challenges. Integration of the clinical history, serologic findings, CSF analysis, and characteristic MRI abnormalities was essential for establishing the correct diagnosis and avoiding inappropriate antiviral treatment. The disappearance of EBV DNA following antibacterial therapy supports the interpretation that viral detection reflected reactivation rather than primary EBV disease. In addition, the patient's unexpected sudden death despite neurological improvement raises concern for occult cardiovascular involvement of late-stage syphilis. Comprehensive cardiovascular and large-vessel evaluation should therefore be considered in patients with advanced syphilis, even when neurological manifestations appear to have stabilized.
